# The Effect of Extremely Low-Frequency Magnetic Field on Stroke Patients: A Systematic Review

**DOI:** 10.3390/brainsci14050430

**Published:** 2024-04-26

**Authors:** Renata Marchewka, Tomasz Trzmiel, Katarzyna Hojan

**Affiliations:** 1 Neurorehabilitation Ward, Greater Poland Provincial Hospital, 60-480 Poznan, Poland; renata_marchewka@wp.pl (R.M.); khojan@ump.edu.pl (K.H.); 2 Department of Occupational Therapy, Poznan University of Medical Sciences, 60-781 Poznan, Poland; 3 Department of Rehabilitation, Greater Poland Cancer Centre, 61-866 Poznan, Poland

**Keywords:** stroke, extremely low frequency electromagnetic field therapy, rehabilitation

## Abstract

Background: The aim of this study was to review the current state of scientific evidence on the effect of extremely low-frequency magnetic fields stimulation (ELF-MFs) on stroke patients. Methods: A systematic review of PubMed, ScienceDirect, PeDro and Embase databases was conducted. Only articles published in English, involving adult participants and focusing on individuals who had experienced a stroke, specifically examining the impact of ELF-MFs on post-stroke patients and had well-defined criteria for inclusion and exclusion of participants, were included. The methodological quality of the included studies was assessed using the Quality Assessment Tool for Quantitative Studies (QATQS). Results: A total of 71 studies were identified through database and reference lists’ search, from which 9 were included in the final synthesis. All included studies showed a beneficial effect of ELF-MFs on stroke patients, however seven of the included studies were carried by the same research group. Improvements were observed in domains such as oxidative stress, inflammation, ischemic lesion size, functional status, depressive symptoms and cognitive abilities. Conclusions: The available literature suggests a beneficial effect of ELF-MFs on post-stroke patients; however, the current data are too limited to broadly recommend the use of this method. Further research with improved methodological quality is necessary.

## 1. Introduction

Stroke is one of the leading causes of death and disability [1,2]. Only in the year 2017 in the European Union, 1.12 million stroke incidents were registered, with 0.46 million deaths caused by stroke. According to an estimation, these numbers will be even higher by the year 2047 [3]. Stroke is a serious condition caused by cerebrovascular disruption of the blood flow. Its impact on patients often leads to a loss of independence, disability and necessity of institutionalization [4]; it also increases the burden on the healthcare system and healthcare providers. The cost of healthcare of stroke patients in Europe in 2010 was estimated to be EUR 64 billion. With increasing aging in societies, its burden on the healthcare system may be even higher [1], which makes stroke one of the most important public health issues.

Currently, there are many methods available for treating stroke patients. Thanks to the application of treatments such as thrombolysis, the mortality rate in strokes, as well as negative effects from the perspective of disability, can often be reduced [5]. However, rehabilitation remains a key element in the therapy of post-stroke patients. Properly conducted rehabilitation maximizes the chances of the patient returning to normal functioning and regaining mobility. Modern neurological rehabilitation is based on the phenomenon of neuroplasticity. Neuroplasticity is the organism’s ability to replace lost connections between individual nerve cells, for example, due to a stroke, by creating new connections. This allows other cells to partially or completely take over lost functions [5,6]. 

In the rehabilitation of stroke patients, various forms of therapy are applied, most of which act through the motor system, stimulating the neuroplasticity of the brain. In addition to conventional exercise therapy, methods supporting neuroplasticity through various physical stimuli are also used. Repetitive Transcranial Magnetic Stimulation (rTMS) is a method widely used in psychiatry and is gaining relevance in rehabilitation [7,8]. This method involves locally stimulating selected areas of the brain using a coil placed on the surface of the skull [9]. It has been shown that rTMS supports the recovery of brain function and enhances neuroplasticity mechanisms. Its effects are confirmed by studies assessing the impact of rTMS on cognitive functions, speech, mood as well as motor skills. It is suggested that rTMS can support traditional physiotherapy and occupational therapy, facilitating the patient in achieving their full potential in recovering functions lost due to a stroke [8,10]. Coils in the shape of the number eight are typically used for this type of stimulation, although initial attempts and studies involving this type of stimulation used a round coil. However, it quickly became apparent that with the use of an 8-shaped coil, it is possible to more precisely stimulate specific chosen areas of the brain [9,11]. 

In recent years, research has also emerged assessing the impact of stimulation with low- and extremely low-frequency magnetic fields (ELF-MFs) on post-stroke patients. However, there is significantly less research on this type compared to studies using rTMS. ELF-MFs include stimulation in the range of 0–300 Hz frequency and have biological effects on tissues. There are data showing that ELF-MFs cause functional changes in muscle, neural tissues and bones [12,13]; some researchers even claim that ELF-MFs may impair the ability of the SARS-CoV-2 virus to replicate and attack host cells [14]. It is indicated that ELF-MFs have a beneficial impact on brain tissue and its functions [15,16,17], suggesting that this stimulation method could be advantageous for patients after a stroke. The potential neuroprotective effects of ELF-MFs may have a significant impact, particularly on cells surrounding the direct focal point of brain damage, located in the so-called penumbra area. The penumbra area is the region surrounding the damage. During a stroke, due to impaired blood flow, mitochondrial function is compromised, leading to disruptions in cellular respiration and oxidative stress. This results in an expansion of the area that may potentially be damaged (the penumbra area), beyond the initially affected region [18]. Research using transcranial magnetic stimulation suggests that it may act as a factor regulating proper mitochondrial function, thereby increasing the chances of survival and restoration of normal function for cells in the penumbra area [19]. However, it is crucial for the exposure to the magnetic field to occur relatively soon after the ischemic episode to most efficiently enable such restoration. According to Capone et al., the action of ELF-MFs also increases the chances of survival for these cells, ensuring more effective neuroplasticity mechanisms, reduced neurological deficits and a faster return to functionality [20]. 

Due to the fact that the majority of studies on magnetic field stimulation in post-stroke patients focus on transcutaneous stimulation, and at the same time, reports are emerging about observed therapeutic benefits of ELF-MFs, it is essential to gather and summarize research assessing the impact of ELF-MFs on post-stroke patients. The aim of this study was, based on the available literature, to answer the question of whether the use of ELF-MFs is beneficial for post-stroke patients.

## 2. Materials and Methods

### 2.1. Study Design and Search Protocol

A review of the literature was conducted in accordance with the Preferred Reporting Items for Systematic Reviews and Meta-Analyses (PRISMA) guidelines [21]. Search strategy and searched databases were established a priori in the review protocol. This review was registered at PROSPERO (CRD42024518320). Two researchers (RM and TT) independently searched the following online data bases: Pubmed, Science Direct, PeDro and Embase. An algorithm with the key “extremely low frequency electromagnetic field” AND “stroke” was used to find suitable publications. Additionally, the reference sections of the relevant literature were manually inspected to identify unique records. The literature review was conducted from 1 March 2024, to 14 March 2024. 

### 2.2. Inclusion/Exclusion Criteria

Inclusion criteria were as follows: randomized controlled trials and other studies involving a control group or pre–post comparison were included if they involved adult participants and focused on individuals who had experienced a stroke. The study had to specifically examine the impact of ELF-MFs on post-stroke patients. Additionally, the research should have included well-defined criteria for both inclusion and exclusion. Studies that involved animals or cell lines, were not published in English or included participants other than those who had experienced a stroke were excluded from this review. Two authors (RM and TT) independently evaluated the identified records for meeting inclusion or exclusion criteria. Discrepancies between the authors’ decisions were resolved by the third author (KH).

### 2.3. Methodological Quality Assesement

All included studies were assessed using the Quality Assessment Tool for Quantitative Studies (QATQS) [22,23], which allows to determine their methodological quality. The QATQS allows to evaluate the methodological quality of studies, and it assesses 8 sections: selection bias, study design, confounders, blinding, data collection methods, withdrawals and dropouts, intervention integrity and analysis. The evaluator classifies the first 6 sections as “weak”, “moderate” or “strong”, according to a reviewer’s dictionary. If any section receives a “weak” evaluation, the overall study is considered “moderate”. If multiple sections are rated as “weak”, the entire study is categorized as “weak”. Conversely, if no section is assessed as “weak”, the study is deemed “strong”. The last 2 sections give additional information about intervention integrity and whether the analysis applied was proper to the scientific question asked. The assessments were performed independently by two researchers (RM and TT). If agreement on the quality assessment could not be reached by the two authors, the third author was consulted (KH).

### 2.4. Data Extraction and Synthesis of the Finding

For detecting duplicates and organizing retrieved records and coordinate screening of the records, Rayyan software (https://www.rayyan.ai, 1–14 March 2024) was utilized. Data regarding authors, title, year, intervention, outcome (changes in the severity of physical, psychological and cognitive functioning and symptoms; changes in laboratory test results), safety and studied population were manually extracted by two authors (RM and TT). A summary encompassing all the discoveries pertinent to the present review was compiled subsequent to an agreement reached by all contributing authors.

## 3. Results

### 3.1. Literature Search and Methodological Quality

**Figure 1:**
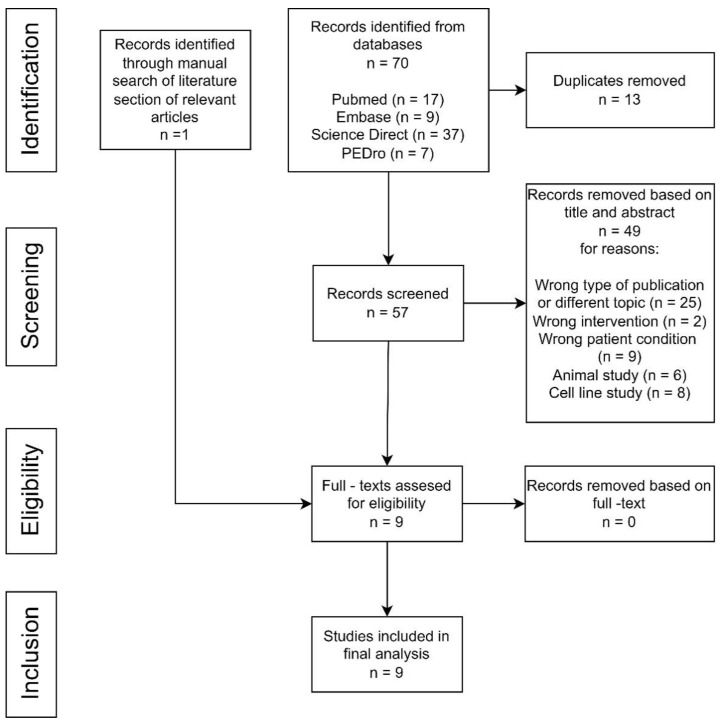
Study flow diagram.

A total of 70 studies were found after the electronic literature search, while one additional study was identified through the reference lists of the relevant articles (Figure 1). Fifty-seven articles remained after duplicates’ removal. After reading the title and the abstract, 49 studies were found to not fulfill the inclusion criteria, matched exclusion criteria or were irrelevant and were excluded from the study from various reasons (wrong type of publication or different topic, wrong intervention, wrong patient condition, animal study or cell line study). All of the remaining nine studies were included after reading the full-text manuscript (Figure 1).

**Figure 2:**
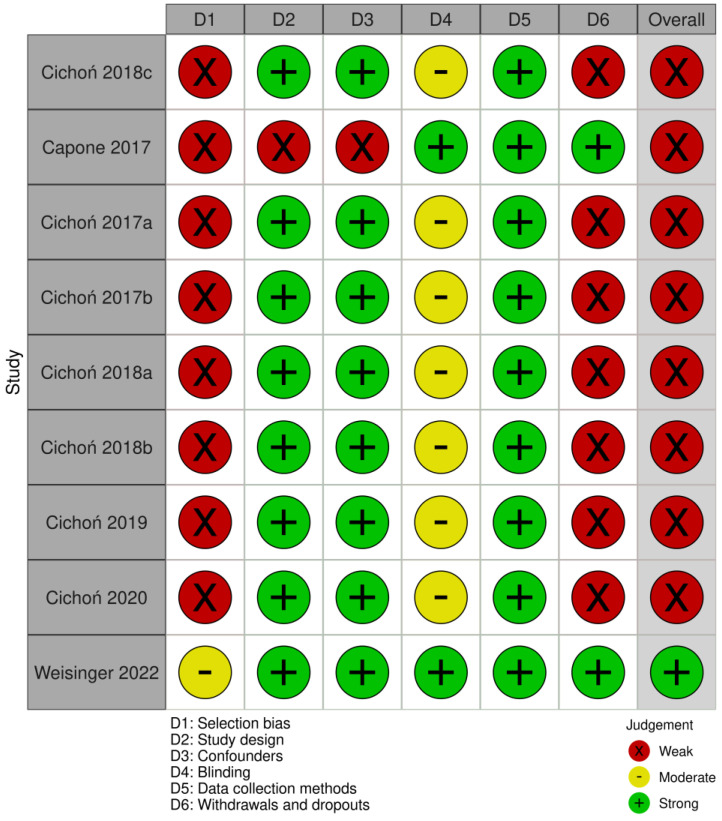
Evaluation of the methodological quality of each study using QATQS [18,22,23,24,25,26,27,28,29].

**Figure 3:**
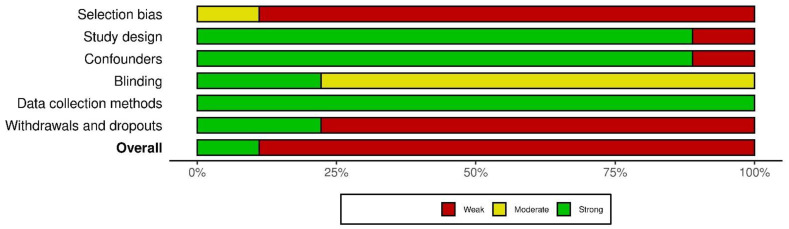
Evaluation of the individual items within the quality sections presented as percentages across all included studies.

Using the QATQS, only one study [24] was deemed to be “strong”, the remaining eight studies [20,25,26,27,28,29,30,31] were assessed as “weak”. Detailed results of the methodological quality assessment for each study are presented in Figure 2. The results for individual sections among all studies showed that “selection bias” and “withdrawals and dropouts section” were assessed as the weakest while the “data collection methods” section was deemed the strongest. In total, 89% (8 out of 9) of the articles were rated as strong in the “study design” and “confounders categories”, whereas in the “blinding” category, 78% (7 out of 9) were rated as moderate due to the lack of description of blinding in the articles (Figure 3). 

### 3.2. Participant Characteristics and Study Design

According to the data regarding participants extracted from included publications, it appeared that the studies were conducted on 381 participants in total. However, five publications [25,26,29,30,31] conducted research on the same group of 48 participants, while in two subsequent publications [27,28], research was also conducted on the same group of 57 participants. Thus, the total number of participants included in the studies was not 381 but rather a group of 132 individuals. Data regarding each study’s participants are presented in Table 1.

Eight studies [24,25,26,27,28,29,30,31] had a randomized controlled trial design with a sham intervention as the control group. One study [20] had two groups—a group exposed to shorter stimulation serving as the control group and a group exposed to longer stimulation. In seven studies, ELF-MFs were applied to the pelvic area [25,26,27,28,29,30,31], in one, head was exposed [20] and in one [24], Electromagnetic Network Targeting Field therapy exposing the entire brain and the cervical and upper thoracic portion of the spine was utilized; in this study, ELF-MF was performed with a device that was utilizing machine learning algorithms to identify spectral patterns characterizing motor functions within EEG measurements, collected during motor tasks.

### 3.3. Results of Therapeutic Interventions

Most of the included studies [25,26,27,28,29,30,31] focused on laboratory tests parameters; some of them also include the measurement of the functional status of the patients. The study conducted by Weisiniger et al. [24] examined the impact of ELF-MFs on stroke patients’ upper limb function and functional status of the patients. The study conducted by Capone et al. [20] was focused on the evaluation of safety and tolerability of ELF-MF patients and examined stroke brain lesions via MRI and the functional status of the patients. Detailed information about measured domains and design including ELF-MF details are presented in Table 2.

#### 3.3.1. Effect of ELF-MFs on Laboratory Tests Measures

Seven of the included studies were conducted by the same group of researchers, and main focus of their study was measuring the effect of ELF-MFs on laboratory test measures. In different publications, they covered a wide set of different biomarkers. They observed a decreased level of carbonyl groups (*p* < 0.001) and an increased level of thiol groups (*p* < 0.01) in plasma proteins of the patients who were rehabilitated using magnetotherapy [28]. The reduction in oxidative stress markers was significantly greater with an increasing number of treatments, and this effect was weaker and non-significant in CG. The level of protein carbonylation was lower in the EG group than in the CG, both after 10 treatments (18% vs. 7%; *p* < 0.05) and after 20 sessions (36% vs. 1%; *p* < 0.001). The authors of that study strongly assert the hypothesis that rehabilitation utilizing ELF-EMF has a beneficial impact on enhancing the psychophysical condition of post-stroke patients. This improvement is associated with a reduction in the levels of in vivo protein oxidative stress parameters. 

In another publication [26], an increased level of 3-nitrotyrosine (*p* < 0.05) in the experimental group was shown. The increase was significantly higher with an increased number of sessions, and it increased more in the experimental group than in the control group after 10 treatments (68% vs. 15% *p* < 0.05). Also, an elevated nitrate/nitrite concentration (*p* < 0.01) in the plasma of patients from the experimental group was observed. The level of nitrate/nitrite in the control group decreased after 10. There were no significant changes and no between-group differences in regard to TNFα concentration or NOS2 mRNA expression after treatments in the studied groups. 

Cichoń et al. [27] also showed that catalase activity in erythrocytes and superoxide dismutase were significantly higher in the experimental group than in the control group. The observed increase in catalase activity after 10 sessions of ELF-MFs was 20% higher than in the control group, while superoxide dismutase showed a twofold higher increase in the experimental group compared to that in the control group (specifically, 40% versus 20%, respectively). In both groups, the change in the total antioxidant status (TAS) of plasma level after both 10 and 20 therapy sessions, with or without ELF-EMF, was low and not statistically significant, which the authors interpreted as a lack of influence of ELF-MF therapy on the antioxidant activity of low-molecular weight antioxidants in the bloodstream. 

In another publication [25], authors proved that ELF-MFs can increase the BDNF level. In the experimental group, the plasma level after ten sessions of rehabilitation incorporating ELF-MFs was significantly higher compared to that in the control group (*p* < 0.0001). The increase in the BDNF level in the experimental group was about 200% (*p* < 0.0001), while in the control group, pre- and post-treatment levels were comparable (*p* > 0.05). In the experimental group, the expression of BDNF in whole blood samples increased about 195% (*p* < 0.0001), while in the control group, it did not change.

Cichoń et al. [29] demonstrated that the level of catalase mRNA expression rose approximately 100% (*p* < 0.01) after ten sessions of rehabilitation involving ELF-MFs. The level of CAT mRNA was significantly higher in the experimental group than in the control group (*p* < 0.001). At the same time, the level of CAT mRNA expression remains at the same level as in the control group. A similar effect was observed in regard to SOD1 and SOD2 mRNA levels. In the experimental group, an increase was noted (>100%, *p* < 0.01 and >200%, *p* < 0.01 for SOD1 and SOD2 mRNA, respectively), while in the control group, these parameters remain unchanged (*p* > 0.05). ELF-MFs impacted also the expression of GPx1 and GPx4 mRNA, which increased in the experimental group about 160% (*p* < 0.001) and 140% (*p* < 0.001), respectively. 

ELF-MFs can influence the expression of some pro-inflammatory cytokines, which was presented in another publication [30]. In this study, the plasma level of IL-1β and the gene expression in whole blood samples of IL-1β in the experimental group after 10 sessions of rehabilitation which involved ELF-MFs were significantly higher than in the control group (*p* < 0.05). There was an increase in the IL1β level and the expression of IL-1β mRNA in experimental group (approximately 100%, *p* < 0.05 and 70%, *p* < 0.001, respectively), while there was no statistically significant change in the control group. A similar effect was observed in regard to the IL-2 plasma concentration, which increased (approximately by 15%, *p* < 0.05) in the experimental group while remaining unchanged in the control group (*p* > 0.05). 

Cichoń [31] showed that in the experimental group, BAX mRNA, CASP8 mRNA, TNFα mRNA and TP53 mRNA expression levels increased after 10 sessions of rehabilitation with ELF-MFs, while in the control group, such a growth was not observed and expression levels of the abovementioned genes remained unchanged. The observed increase was approximately 50% (*p* < 0.01) for CASP8 mRNA, 100% (*p* < 0.001) for BAX mRNA, 50% (*p* < 0.001) for TNFα mRNA and 100% (*p* < 0.001) for TP53 mRNA. No such growth was showed in regard to BCL-2 mRNA in either group.

#### 3.3.2. Effect of ELF-MFs on Functional Status and Upper Limb Function and Ischemic Lesion

Capone et al. [20] showed that clinical conditions improved similarly in all the patients in both ELF-MFs groups in regard to the National Institute of Health Stroke Scale, Barthel index and modified Rankin scale scores. In all the patients who were stimulated for 120 min, a reduction in the ischemic lesion was observed in the 1 month follow-up, while in the group stimulated for 45 min, the lesion volume increased in two patients and reduced in one. It is worth noting that despite no adverse effects of 45 min and 120 min of stimulation, 240 min of stimulation (which was planned by the authors) was not performed because of the non-acceptance of the participants and their physicians.

In the study conducted by Weisiniger et al. [24], in regard to the primary outcome, participants from the experimental group noted a higher increase. In the experimental group in week 4, the mean score of Fugl-Meyer Assessment—Upper Extremity was 23.2 ± 14.1 and 31.5 ± 10.7 in week 8, while in the same timeframes in the control group, scores were 9.6 ± 9.0 and 23.1 ± 14.1, respectively, for week 4 and 8 with no statistical differences between both groups at the baseline of the study. A similar effect was observed for secondary outcomes, where higher improvement occurred in the experimental than in the control group. Only the scores of Patient-Reported Outcome Measurement Information System Global 10 did not differ between groups at 4 and 8 weeks. 

Cichoń et al. [28] observed a beneficial impact of using MLF-EMs in the rehabilitation process. In their study, the ADL value in patients treated with ELF-EMF increased by 25% compared with that in the control group (*p* < 0.01). They also observed an increase in MMSE, which constituted improvement, and a decrease in GDS, which also should be treated as improvement (improvement in comparison to the control group was 35%, *p* < 0.05, and 65%, *p* < 0.001, for MMSE and GDS, respectively). This was also reported in other included publications prepared by this group of researchers, as they conducted various laboratory blood tests on the same group of patients [25,26,27,29,30,31].

## 4. Discussion

The objective of this systematic review was to assess, based on the available literature, the impact of ELF-MFs on patients post stroke. The literature findings suggest favorable effects of such interventions on post-stroke patients. The majority of the available literature considering using electromagnetic fields stimulation in the rehabilitation of stroke survivors are focused on rTMS, thus only nine studies of ELF-MFs in stroke patients have been identified in the present systematic review. Most of the included studies were focusing on examining the effect of MLF-MFs on the changes in biomarkers such as BDNF levels, oxidative stress markers and inflammation markers [25,26,27,28,29,30,31]. 

In the majority of the articles included in this study, a neuroprotective effect of ELF-MFs was indicated. Three of the studies [27,28,29] presented an antioxidant effect in the form of decreased level of carbonyl group in the experimental group [28], increased activity of antioxidant enzymes (superoxide dismutase and catalase) [27] or increased mRNA expression of those enzymes’ genes [29]. Similar antioxidant results were obtained by Medina-Fernandez et al., who conducted a study on the effects of rTMS on rats [32]. In their study, rats were injected with a myelin oligodendrocyte protein to create a multiple sclerosis-like model. The authors observed reduced oxidative stress markers in rats treated with rTMS. Medina-Fernandez et al. attributed this antioxidant effect to the beneficial effect of rTMS on mitochondrial activity (in their study, there was a 67.7% decrease in mitochondrial activity in the control group, while in the experimental group, this decrease was only 7.1%). In another study conducted by Cichoń et al. [26], the authors presented increased NO levels as the effect of ELF-MF stimulation and pointed its neuroprotective role through blood flow regulation. Another study included in this review also indicates the neuroprotective effects of ELF-MFs. Capone et al. [20] attribute the observed reduction in stroke lesions to the regulation of adenosine receptors. One hypothesis explaining such neuroprotective and regenerative effects of ELF-MFs suggests that the stimulation is perceived by the body and its cells as a low-intensity stressor, triggering the release of neuroprotective and regenerative factors such as BDNF and antioxidants [33]. Other authors more precisely point to specific mechanisms, for example, the mechanism of action of ELF-MFs in this process may be explained by the increased influx of calcium ions through L-type voltage-gated calcium channels, leading to an increased expression of BDNF mRNA [34]. Due to the ambiguity and differing hypotheses regarding the mechanisms of action of this form of stimulation, it is likely that multiple different processes are occurring simultaneously, contributing to the observed changes. In a different study conducted by Cichoń et al. [31], the researchers discovered that exposure to ELF-MFs significantly elevated the expression of pro-apoptotic genes (BAX, CASP8, TNFα, TP53) in post-stroke patients, potentially indicating the activation of signaling pathways related to brain plasticity processes; however, they stated that additional research is required for a more comprehensive understanding of this phenomenon. ELF-MFs effect on apoptosis of human cells has been previously reported by other researchers. Ding et al. [35] discovered that simultaneous exposure of cells to H_2_O_2_ and ELM-MFs significantly increased the number of apoptotic and necrotic cells compared to cells treated with H_2_O_2_ alone. Analysis of BAX protein levels showed no significant differences between H_2_O_2_-treated cells and those concurrently exposed to the magnetic field. Although the magnetic field alone did not induce apoptosis or necrosis, it enhanced the process associated with H_2_O_2_-induced cell death. It is worth noting that ELF-MFs used in the research conducted by Ding et al. had different parameters (24 h exposure to 60 Hz, 5 mT) and were conducted on HL-60 leukemia cell lines. Contrary to this finding, a study conducted on the LAN-5 neuroblastoma cell line by Pirozzoli et al. [36] showed that 24 h exposure to 50 Hz, 1 mT ELF-MFs significantly weakens apoptosis induced by camptothecin in this cell line. As indicated by Deng et al. [37], both apoptosis and autophagy are neuroprotective processes in the context of ischemic stroke. Both of these processes occur intensively, especially in the first hours after the onset of the vascular incident. Most likely, these processes limit the expansion of the stroke focus into the penumbral area, exerting a neuroprotective effect. Therefore, supporting and regulating these processes appear crucial to ensure the maximum recovery of patients after ischemic stroke. However, as Deng et al. point out, it has not been conclusively determined how these processes should optimally proceed and whether and how they can be influenced with the help of ELF-MFs after the acute phase of the stroke. 

Neuroplasticity involves both the formation of new synapses and the generation of new nerve cells, as well as neuroprotection [38]. Both neuroplasticity and neuroprotection, as well as neurotrophism, are processes that occur simultaneously, complementing and interpenetrating each other, often regulated by similar mechanisms, processes and chemical substances [39,40]. Inflammation has both neuroprotective and neuroplasticity promoting effects by stimulating trophic factors [41]. IL-1β is a good example of a substance with both neuroprotective and neuroplasticity promoting effects. Cichoń et al. [30] also showed that ELF-MFs modify the inflammatory response of the organism, increasing the levels of certain cytokines (IL-1β and IL-2). At the same time, they suggest that heightened levels of the inflammatory response may have beneficial effects in the case of stroke patients. They indicate that the neuroprotective effect of IL-1β they describe may be associated with the activation of neurotrophic factors in response to the increased level of IL-1β. However, this hypothesis is not aligned with the findings presented by other researchers [42], who showed that IL-1 is a main contributor to ischemic brain damage and IL-1 gene deletion results in less brain damage during ischemia. Studies regarding the effects of IL-1β on stroke patients are not conclusive; they indicate both its inflammatory effect, contributing to the increase in the area of damage during a stroke, and its positive effects in terms of neuroprotection and neuroplasticity. Most likely, the difference between the damaging and positive effects of IL-1β arises from its local concentration and the actions of additional factors that control and modulate its effects. The increase in BDNF concentration and BDNF mRNA expression observed in the Cichoń et al. study [25] indicates the neuroplasticity and regenerative effects of ELF-MFs on patients after stroke. These findings are in line with the results of the study by Urnukhsaikhan et al. [43] conducted on mice, which also showed an increase in BDNF concentration following magnetic field stimulation. It is worth mentioning that in the publications included in this review, biomarkers are often presented in the context of both their neuroprotective effects and their promotion of neuroregeneration and neuroplasticity (this is most likely due to the previously mentioned close relationship between the phenomena of neuroplasticity and neuroprotection). These biomarkers and their influence on neuroplasticity are also attributed to the observed improvement in clinical indicators of the patient’s condition, such as cognitive abilities or motor functions. 

An improvement in cognitive abilities reported in some of the included studies [25,26,27,28] is in line with other researchers’ finding. It is reported that ELF-MFs can modulate the activity of the human brain cortex [44]. Meta analysis performed by Barth et al. [45], which included nine studies, showed a limited significant positive effect of ELF-MFs on the cognitive abilities of exposed subjects; moreover, studies on rats [46] and monkeys [47] also showed that ELF-MFs improved the cognitive abilities of those animals. The observed improvement in cognitive abilities due to ELF-MF stimulation may result from a facilitation of forming new neurons in the hippocampal area, as demonstrated in studies on rats [46].

An improvement in depressive symptoms was also reported in the same number of the included studies [25,26,27,28]. Contrary to those results, exposition on ELF-MFs in the occupational environment may increase depression severity [48]. However, this kind of exposition differs from the exposition used in the studies included in this systematic review; the main difference is the time of the exposition, which in the case of environmental ELF-MFs, studied in the research conducted by Bagheri Hosseinabadi et al., was 8 h a day. Differences are noted between short-term and chronic exposure to magnetic fields. The initial beneficial effects of short-term stimulation may diminish as the stimulation becomes chronic [44]. The observed improvement in GDS scores in the abovementioned included studies may be a result of modulating the inflammatory response, as well as modulating regeneration-related processes, such as an increased expression of BDNF. Another important aspect is that the studies included in this review, which demonstrate improvement in depressive symptoms, were rated by us as moderate in terms of blinding because they did not describe whether blinding of participants or researchers was present, and if so, how it was implemented. This may indicate that researchers were aware of the study’s purpose and group allocation, potentially introducing bias in the assessment of depression symptoms improvement.

Five of the included studies showed improvement in motor function or the functional status of patients exposed to ELF-MFs [24,25,26,27,28]; these findings are coherent with those in the literature regarding various forms of magnetic stimulation, which are reported to reduce spasticity [49], improve independence in activities of daily living [50,51], improve hand and/or upper extremity function [52,53,54,55]. All the studies by Capone et al. [20], Cichoń et al. [25,26,27,28] and Weisiniger et al. [24] demonstrated a significantly greater improvement in ADLs in the groups receiving ELF-MFs compared to that in the control group (or the group with a shorter exposure time in the case of Capone et al. [20]). Cichoń et al. [27] suggest that such results may be related to the pro-antioxidant effect of ELF-MFs, as oxidative stress negatively impacts brain plasticity, which hinders motor recovery and contributes to the progressive decline in cognitive abilities, which can significantly influence independence in ADL. Factors other than motor functions and cognitive abilities, such as pain, spasticity or nutritional status, also influence ADL [56,57,58]. However, these factors were not investigated in the studies included in this review, therefore caution should be exercised in interpreting the presented results.

The mechanism underlying the improvement in upper limb function presented by Weisinger et al. [24] is uncertain. It is possible that the beneficial effect of ELF-MFs on neuroplasticity may be at the core, including mechanisms discussed earlier in this review, such as the regulation of L-type voltage-gated calcium channel function or BDNF expression.

Particularly noteworthy is the fact that none of the studies included in this review reported any negative side effects of ELF-MFs. A review of in vivo experiments conducted in 2023 [59] indicated that the majority of studies suggest that exposing patients to ELF-MFs does not result in significant adverse outcomes, thus this therapy method is deemed safe. However, reports presenting undesirable effects of ELF-MFs can be found in the literature. Zwolińska et al. [60], in a study on the effectiveness of this therapy form in patients with rheumatoid arthritis, observed adverse effects in 5 out of 39 study participants. It is worth noting that four of these adverse effects occurred in the group exposed to extremely low-frequency pulse magnetic fields. In the group exposed to static magnetic field, adverse effects were observed in only one person. The adverse effects reported by the authors include insomnia, heart palpitations, headaches and exacerbation of rheumatoid arthritis symptoms. Thamsborg et al. [61] also reported adverse effects of such stimulation in their patients, however these adverse outcomes were mostly the feeling of warmth or pulsation; few patients reported an exacerbation of osteoarthritis symptoms. The results of our review remained in line with those from most of the literature claiming that ELF-MFs is a safe form of treatment.

Most studies indicate effectiveness in improving laboratory markers related to neuroplasticity, inflammation and antioxidant activity. Moreover, authors primarily highlight improvements in the clinical indicators of stroke survivors. Even though there is no clear explanation for the processes underlying the improvement in cognitive abilities, independence in daily activities, motor function or depressive symptoms, these improvements have a significant impact on the quality of life of patients. The above data suggest that ELF-MFs may play a significant supportive role in the rehabilitation process of stroke patients. However, in the interpretation of these results, it is crucial to consider several significant factors which may be considered as limitations. The majority of studies included in this literature review were conducted using Extremely Low-Frequency Magnetic Fields (ELF-MFs) targeted at the pelvic region. This might seem surprising considering that the therapy was administered to patients with brain damage resulting from a stroke, so the head and the brain seem to be the target of the therapy. In the studies conducted by Cichoń and colleagues, this approach was adopted, because they claimed that exposure of the head to MLF-EMs can result in the activation of the epilepsy focus in brain [26]. However, the authors did not back this statement with a citation or link to any study confirming this statement. Capone et al. [20] conducted their study exposing the head and reported no adverse outcomes. It is worth noting that the time of the exposition in their study was up to two hours but induction was lower than in the studies conducted by Cichoń et al. This safety study was also conducted on a very limited number of participants. However, the statement that ELF-MFs may induce epilepsy seems to lack support from the data. This statement seems even more controversial in the light of the fact that magnetic stimulation in the form of transcranial magnetic stimulation of the brain is considered as a useful tool in the treatment of epilepsy [62]. This stimulation can be used to stop ongoing seizure as well as to prevent it from occurring when it is used between seizures. Up to date, there are no studies comparing the effect of the head versus pelvic exposition to ELF-MFs, thus such studies should be conducted in the future.

Another important aspect of the studies included in this systematic review is that majority of the studies are conducted by the same research team on the same group of stroke survivors [25,26,27,28,29,30,31], which may dampen their generalization possibility. Finally, the third significant factor is that the number of available studies on this topic remains limited; moreover, the available studies were conducted on small patient groups, most of which also have methodological shortcomings that may significantly impact the risk of bias. It is conceivable that utilizing keywords strictly focused on MLF-MFs in the search to locate publications more aligned with MLF-MFs than rTMS (which was not within the scope of interest of this review) might have led to some publications concerning the application of MF-MFs not being identified.

## 5. Conclusions

The available literature suggests a beneficial effect of ELF-MFs on post-stroke patients; however, caution is advised in interpreting these results, particularly in their translation to clinical practice, as the current data are too limited to broadly recommend the use of this method. Further research with improved methodological quality is necessary.

## Figures and Tables

**Table 1:** Characteristics of the participants of the included studies.

First Author and Year	N of Participants	Age	Inclusion Criteria	Exclusion Criteria
Capone 2017a [20]	6 patients (3 in 45 min stimulation group, 3 in 120 min stimulation group)	76.3 ± 6.1	>18 years with first mono-hemispheric ischemic stroke, onset of symptoms within 48 h and NIHSS score > 4	Acute intracranial hemorrhage; previous ischemic or hemorrhagic stroke; history of seizure; contraindications to magnetic field exposure; life expectancy < 3 months; other serious illness or complex disease that may confound treatment assessment; women known to be pregnant, lactating or having a positive or indeterminate pregnancy test; simultaneous participation in another study
Cichoń 2017a [26]	48 (25 in EG and 23 in CG)	48.8 ± 7.7 in EG, and 44.8 ± 8.0 in CG	Post-stroke patients with moderate stroke severity according to NIHSS score	Patients with metal and/or electronic implants
Cichoń 2017b [27]	57 (23 in EG and 34 in CG)	68.0 ± 15.8 in EG, 70.9 ± 15.3 in CG	Agreeing to participation, moderate stroke severity according to NIHSS	Patients with metal and/or electronic implants
Cichoń 2018a [25]	48 (25 in EG and 23 in CG)	48 ± 8 in EG, and 44.8 ± 7.7 in CG	Agreeing to participation, moderate stroke severity according to NIHSS	From EG and CG: neurological illness other than stroke, hemorrhagic stroke, chronic or significant acute inflammatory factors, dementia, and/or decreased consciousness in their medical pre-stroke history. Only from EG: electronic and/or metal implants
Cichoń 2018b [29]	48 (25 in EG and 23 in CG)	48 ± 8 in EG, and 44.8 ± 7.7 in CG	2–4 weeks after ischemic stroke	From EG and CG: hemorrhagic stroke, neurological illness other than stroke, dementia, chronic or significant acute inflammatory factors. Only from EG: electronic and/or metal implants
Cichoń 2018c [28]	57 (23 in EG and 34 in CG)	68.0 ± 15.6 in EG, 70.9 ± 15.3 in CG	Agreeing to participation, moderate stroke severity according to NIHSS	Patients with metal and/or electronic implants
Cichoń 2019 [30]	48 (25 in EG and 23 in CG)	48 ± 8 in EG, and 44.8 ± 7.7 in CG	Ischemic stroke	Neurological illness other than stroke, other types of stroke than ischemic stroke; chronic or significant acute inflammatory factors; and/or dementia
Cichoń 2020 [31]	48 (26 in EG and 22 in CG)	48 ± 8 in EG, and 44.8 ± 7.7 in CG	Moderate stroke severity, 3–4 weeks after incident	From EG and CG: chronic or significant acute inflammatory factors, dementia, hemorrhagic stroke, neurological illness other than stroke, and/or decreased consciousness in their medical pre-stroke history. Only from EG: metal and/or electronic implants
Weisinger 2022 [24]	21 (13 in EG and 8 in CG)	54.3 ± 17.8 in EG, and 55.3 ± 10.1 in CG	Patients 4–21 days post-ischemic stroke with first stroke or no prior upper extremity impairment, right hand dominant, with a FMA-UE score between 10 and 45.	Patients who were not medically stable, with a physiological, neurological or psychiatric history that might confound study measures or contraindications for MRI scanning

EG—experimental group, CG—control group, NIHSS—National Institutes of Health Stroke Scale, MRI—magnetic resonance imaging, FMA-UE—Fugl-Meyer Assessment—Upper Extremity.

**Table 2:** Intervention and measured characteristics of the included studies.

First Author and Year	Type of Intervention and Control	Magnetic Stimulation Parameters	N of Sessions	Additional Therapies	Measured Outcome	Measurement Tools
Capone 2017 [20]	First three patients were stimulated for 45 min/day and the following three patients for 120 min/day	Single-pulsed signals at 75 ± 2 Hz, with a pulse duration of 1.3 and peak intensity 1.8 ± 0.2 mT. ELF-MF was applied to head by flexible coil, upon the ischemic hemisphere.	5	n/d	Stroke lesion volume, clinical condition of the patients	Volumetric changes in the stroke lesions (calculated based on MRI scans), NIHSS; Barthel index; modified Rankin scale
Cichoń 2017a [26]	EG-ELF-MF, CG—placebo exposure to ELF-MF	EG patients were exposed for 15 min (frequency—40 Hz, magnetic induction—7 mT), ELF-MF was applied to pelvic girdle.	20	Rehabilitation program consisting of aerobic exercise for 30 min, neurophysiological methods for 60 min and psychological therapy for 15 min.	Nitric oxide generation and its metabolism, functional status	Level of 3-NT and nitrate/nitrite concentration in the plasma, TNFα, ADL, GDS and MMSE
Cichoń 2017b [27]	EG-ELF-MF, CG—placebo exposure to ELF-MF	EG patients were exposed for 15 min (frequency—40 Hz, magnetic induction 7 mT, waveform—bipolar, rectangular). ELF—MF was applied to pelvic girdle.	20	Rehabilitation program consisting of aerobic exercise for 30 min, neurophysiological methods for 60 min, and psychological therapy for 15 min.	Oxidative stress, functional status	Catalase activity estimation, superoxide dismutase estimation, determination of TAS, ADL, GDS and MMSE.
Cichoń 2018a [25]	EG-ELF-MF, CG—placebo exposure to ELF-MF	EG patients were exposed for 15 min (frequency—40 Hz, magnetic induction—5 mT, waveform—bipolar, rectangular). ELF-MF was applied to pelvic girdle.	10	Rehabilitation program consisting of aerobic exercise for 30 min, neurophysiological methods for 60 min and psychological therapy for 15 min.	Growth Factors involved in the neuroplasticity process, functional status	BDNF Expression in whole blood and in plasma, VEGF in plasma, level of HGF, SCF, SDF-1α, β-NGF, and LIF, ADL, GDS, MMSE, modified Rankin scale.
Cichoń 2018b [29]	EG-ELF-MF, CG—placebo exposure to ELF-MF	EG patients were exposed for 15 min (frequency—40 Hz, magnetic induction—5 mT, waveform—bipolar, rectangular). ELF-MF was applied to pelvic girdle.	10	Rehabilitation program consisting of aerobic exercise for 30 min, neurophysiological methods for 60 min and psychological therapy for 15 min.	Antioxidant enzyme gene expresion	CAT mRNA, SOD1 mRNA, SOD2 mRNA and GPx mRNA expression
Cichoń 2018c [28]	EG-ELF-MF, CG—placebo exposure to ELF-MF	EG patients were exposed for 15 min (frequency—40 Hz, magnetic induction—5 mT, waveform—bipolar, rectangular). ELF-MF was applied to pelvic girdle.	20	Rehabilitation program consisting of aerobic exercise for 30 min, neurophysiological methods for 60 min and psychological therapy for 15 min.	Changes in the level of oxidative stress markers, independence in activities of daily living, cognitive abilities, depression symptoms	TBARS, thiol groups, and carbonyl groups, ADL, MMSE, GDS.
Cichoń 2019 [30]	EG-ELF-MF, CG—placebo exposure to ELF-MF	EG patients were exposed for 15 min (frequency—40 Hz, magnetic induction—5 mT, waveform—bipolar, rectangular). ELF-MF was applied to pelvic girdle.	10	Rehabilitation consisting of aerobic exercise for 30 min, neurophysiological methods for 60 min, in the control group consisted of a 60 min session in the morning (30 min of shaping techniques and 30 min of balance training), 30 min aerobic training (2–3 times a day for 10 min at 60 min intervals) and 30 min muscle strengthening exercises and psychological therapy for 15 min.	Inflammatory cytokines	Levels of IL-1β, IL-2, INF-γ, TGF-β, level of IL-1β mRNA expression.
Cichoń 2020 [31]	EG-ELF-MF, CG—placebo exposure to ELF-MF	EG patients were exposed for 30 min (frequency—40 Hz, magnetic induction—5 mT, waveform—bipolar, rectangular). ELF-MF was applied to pelvic girdle.	10	Rehabilitation program consisting of aerobic exercise for 30 min, neurophysiological methods for 60 min and psychological therapy for 15 min.	Expression level of genes involved in apoptosis	BAX mRNA, BCL-2 mRNA, CASP8 mRNA, TNFα mRNA, TP53 mRNA expression levels.
Weisinger 2022 [24]	ELF-MF technique, brain computer interface-based (BCI-based), low frequency, low intensity, frequency-tuned EMF therapy, ENTF therapy	Non-invasive, frequency-specific, extremely low-frequency (1–100 Hz), low intensity (<1 Gauss) electromagnetic field treatment targeted the participant’s central nervous system, ELF-EMF emission was used solely for the EG group, not the CG. EMF was applied to head and the cervical and upper thoracic spine.	40	3 sessions a week, 10 min of upper extremity physical therapy/occupational therapy-based exercises (e.g., gripping a ball, reaching) participants also received 1 h/day	Change in upper extremity motor function, functional status	FMA-UE, action research arm test; box and blocks test; FMA-LE; modified Rankin scale; NIHSS; Patient-Reported Outcome Measurement Information System Global 10.

ELF-MF—extremely low frequency magnetic field, EG—experimental group, CG—control group, NIHSS—National Institutes of Health Stroke Scale, ADL—Activity Daily Living, GDS—Geriatric Depression Scale, MMSE—Mini Mental State Examination, 3-NT—3-nitrotyrosine, TNFα—tumor necrosis factor alpha, TAS—Total antioxidant status, BDNF—Brain-derived neurotrophic factor, FMA-UE—Fugl-Meyer Assessment—Upper Extremity, FMA-LE—Fugl-Meyer Assessment—Lower Extremity, IL-1β—interleukin 1β, IL-2—interleukin 2, INF-γ—interferon-γ, TGF-β—trans-forming growth factor β, TBARS—thiobarbituric acid reactive substances, HGF—hepatocyte growth factor, VEGF—vascular endothelial growth factor, SCF—stem cell factor, SDF-1α—stromal cell derived factor 1 alpha, β-NGF—beta nerve growth factor, LIF—leukemia inhibitory factor, ENTF—electromagnetic network targeting field therapy.

## Data Availability

No new data were created during this systematic review.
